# “I didn’t really fit into any boxes”: understanding the experiences of women affected by cancer in pregnancy and up to one-year postpartum—a mixed-method systematic review

**DOI:** 10.1007/s11764-024-01695-z

**Published:** 2024-10-26

**Authors:** Lucy Armitage, Marjorie Atchan, Deborah Davis, Murray R. Turner, Catherine Paterson

**Affiliations:** 1 University of Canberra, Faculty of Health, Canberra, Australia; 2 Flinders University, Caring Futures Institute, Adelaide, Australia; 3 Central Adelaide Local Health Network, Adelaide, Australia

**Keywords:** Cancer care, Gestational cancer, Maternity care, Pregnancy, Postnatal, Women’s experience

## Abstract

**Purpose:**

Little is known about women’s experiences of cancer during pregnancy and up to one-year postpartum. As the incidence of gestational cancer rises parallel to increasing rates of early onset cancers there is an imperative need to understand their experiences. The aim of this research is to understand women’s experiences of gestational cancer during pregnancy and up to one-year postpartum.

**Methods:**

This systematic integrative review followed the JBI methodology for mixed method systematic reviews (MMSR) which integrates empirical data from qualitative and quantitative primary studies. The search strategy included electronic databases, APA PsycINFO, CINHAL, Medline, Scopus, and the Web of Science Core Collection. The review has been reported following the Preferred Reporting Items for Systematic Reviews and Meta-Analyses (PRISMA) guidelines. A comprehensive methodological quality assessment was undertaken using the Mixed Methods Appraisal Tool (MMAT).

**Results:**

Thirteen studies were included, reporting on the experiences of 266 women. The findings represented the women’s insight on the psychological impact of their gestational cancer, the impact on women’s identity as a mother and a patient, and women’s experiences of complex care.

**Conclusions:**

Gestational cancer reflects an emerging focus of clinical practice and an opportunity for much needed new research to explore woman-centered care exploring supportive care needs and models of maternity care.

**Implications for Cancer Survivors:**

Women’s experiences indicate that services are under-resourced to address the holistic and integrated supportive care needs of women affected by cancer across both maternity and cancer care teams.

**Supplementary Information:**

The online version contains supplementary material available at 10.1007/s11764-024-01695-z.

## Introduction

Women affected by cancer in pregnancy, and up to one-year postpartum, face two transformative and paradoxical life experiences simultaneously, bringing a new life into the world and facing their own mortality. A woman’s diagnosis and treatment of gestational cancer presents significant challenges alongside pregnancy, childbirth, and parenting [1, 2]. The prevalence of gestational cancers has a reported incidence rate of one in 1000 during pregnancy, with the incidence increasing during the postpartum period, underscoring that it is a growing global health issue which requires attention [3]. For example, a 2023 Australian-based population study reported 33.6 in 100,000 birthing women were affected by gestational cancer during pregnancy, and 99.1 in 100,000 were diagnosed in the postpartum period [2]. Current data reveals an increased incidence of gestational cancers by 2.1% per year and 1.5% increase per year in postpartum cancers since 1994.

Gestational cancers affect women of reproductive age. The most common cancer types diagnosed include breast, cervical cancer, hematological cancers, and multiple myeloma. However, all cancer types may be diagnosed through the perinatal period [1, 4]. Each type of cancer and its associated treatments have entity-specific negative consequences on the health and well-being of the mother, fetus, and their loved ones.

Gestational cancers require a multidisciplinary integrative approach to care provision. Balancing maternal and fetal well-being while determining treatment pathways for optimal clinical outcomes is a highly complex process. There are multiple physical and psychological considerations that include the type of tumor, the pregnancy trimester and gestational age of the fetus, treatment pathway, and the woman’s wellbeing [5, 6]. Currently, there is a paucity in research that reports on woman’s experiences of gestational cancer beyond clinical biomedical outcomes [7]; thus, little is known about their unique holistic experiences. Recognizing the rising incidence of gestational cancers and considering its impact on the experiences of women, further research is warranted. The aim was to understand women’s experiences of gestational cancer during pregnancy and up-to-one-year postpartum.

## Methods

### Design

The study design used inductive analysis, through an interpretive paradigm that established the focus to enable a comprehensive understanding of women’s experiences of gestational cancer [8].

This mixed method review followed the JBI methodology for mixed method systematic reviews (MMSR) which integrates empirical data from qualitative, quantitative and mixed method primary studies. This approach was employed for its ability to effectively summarize and critically synthesize existing research into one comprehensive evidence-based review [9]. The benefit of this method is that it enables a comprehensive synthesis of findings. These findings are reported through numerical data and narrated perspectives that inform a depth of understanding. MMSR is well suited for healthcare research. It gains knowledge on outcomes and experiences to inspire future research that will inform policy and practice and support tailored models of care. The MMSRs used a convergent integrated design to combined data from the qualitative and quantitative studies in a process of data transformation [10].

The review has been reported following the Preferred Reporting Items for Systematic Reviews and Meta-Analyses (PRISMA) guidelines [11], and the review protocol was registered with PROSPERO [CRD42023430210].

### Search strategy and pre-determined eligibility criteria

Database searches were run in in March 2024 with the support of specialist librarian MT. The search strategy included the electronic databases, APA PsycINFO, CINHAL, Medline, Scopus, and the Web of Science Core Collection. To increase the breadth of the database searches, there was no date range set. For a full record of the search strategy, see Supplementary Table 1. In addition to the database searches, the reference lists of all included articles after full text screening were scanned to source additional original studies.

#### Eligibility criteria

Articles were screened according to the inclusion and exclusion criteria outlined in Table 1.

**Table 1:** Inclusion and exclusion criteria

Inclusion	Exclusion
All original research studies irrespective of design	Reviews of the literature
Peer-reviewed literature	Non-peer reviewed literature Editorials Commentaries Commercial websites Blogs
Childbearing women diagnosed with and treated for cancer	Childbearing women without a diagnosis of cancer and without any other complexities affecting pregnancy, birth, or postnatal
Women’s experiences reported	Only clinical treatment pathways reported Only morbidity and/or mortality outcomes reported

### Screening process

All identified studies were imported into Endnote referencing software and exported to Covidence Systematic Review software [12] for the removal of duplicates and to manage the article screening process. Articles were reviewed by two reviewers, LA and CP, according to the eligibility criteria, and any conflicts were resolved by discussion. Full-text articles were reviewed by LA and CP with any disagreements resolved by discussion until consensus was reached.

### Data extraction

Data extraction was performed by one author (LA) using tables piloted on several full-text studies and was carefully quality checked by a second reviewer (CP) where any additional information was added or removed. A summary table was used to extract key study details including purpose, setting, sample size, participant, and study design, then followed by the mixed method data extraction which allowed for qualitative and quantitative data to be formatted into a data extraction table. Mixed method data was extracted into a qualitative data extraction table where individual findings were recorded as direct quotations or interpreted results were identified and ranked using ConQual ranking of either “unequivocal” (clear association between the finding and illustration), “credible” (unclear association between the finding and illustration, leaving it open to challenge), or “not supported” (findings not supported by data). Only unequivocal and credible findings were included in the final synthesis according to the JBI methods [13]; see Supplementary Table 2. Throughout the data extraction and review process, LA and CP discussed each queried element until an agreement was reached to ensure quality in the process. The qualitative, quantitative, and mixed method studies included in this systematic integrative review were extracted in a convergent integrated design. The findings were coordinated in a consistent approach according to the JBI manual for MMSR [10].

### Quality assessment

The methodological quality and evaluation of the studies were assessed using the Mixed Methods Appraisal Tool (MMAT) [14] as it enables qualitative, quantitative, and mixed method research designs to be evaluated using the same form which increases consistency of findings. Methodological quality assessment was reviewed by LA with second quality check by CP. The MMAT used quality appraisal questions relevant to study type and assessed as yes, no, or unclear.

### Data synthesis

As a MMSR, using the JBI methodology [10], data was synthesized sequentially, using a convergent integrated approach enabling succinct integration of both quantitative and qualitative evidence. We applied inductive interpretation to quantitative data where we extracted the data and converted it to a textual description. The process of data transformation is known as qualitizing and subsequently enables integration with qualitative data [10]. Qualitative data were extracted as direct quotations. Findings were then coded, categorized where two or more findings were identified and then refined into themes as identified. This approach enabled a comprehensive structure to describe the articulated findings. The process of data synthesis was carried out by LA and quality checked by CP and DD; the review was facilitated in discussion and conflicts resolved and agreed on to form consensus.

## Findings

**Fig. 1:**
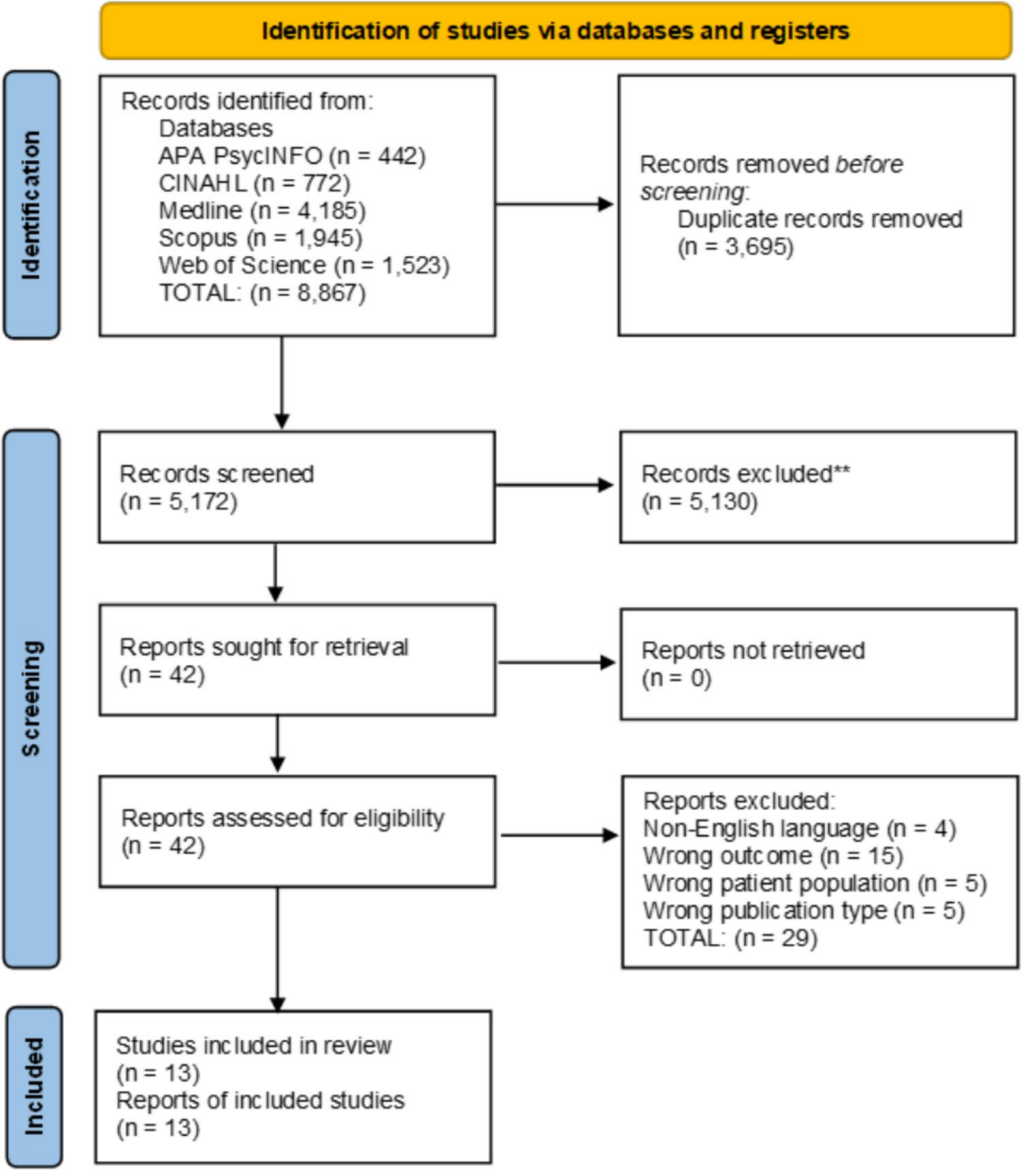
PRISMA diagram

A total of 8867 studies were identified by the combined database searches. After the removal of duplicates, 5172 records were screened by title and abstract, excluding 5130 studies. Forty-two studies were reviewed in full, resulting in the exclusion of a further 29, with reasons, see Supplementary Table 3. A total of 13 studies were included; see Fig. 1.

The included studies highlight an emerging research focus on women’s experiences of gestational cancer, that expands the research beyond clinical treatment pathways and outcomes. The included studies comprised of 10 qualitative studies, two quantitative and one mixed method study [15–27]. The data were collected in middle to high income nations that included Australia (*n* = 3), Italy (*n* = 2), France (*n* = 1), The Netherlands and Belgium (*n* = 1), UK (*n* = 1), US (*n* = 1), Canada (*n* = 1), Brazil (*n* = 1), Japan (*n* = 1), and Singapore (*n* = 1), which was representative of varying healthcare systems and cultural variances. There was a total of 266 women included in the review who were diagnosed with cancer during pregnancy or postnatally. A summary of the included studies is included as Table 2; the results of the methodological quality assessment are presented in Table 3.

**Table 2:** Summary table of included studies

Author (year) and country	Purpose	Setting	Sample size	Participants	Response rate	Attrition	Design	Time points
[15] Italy	To explore women’s experiences of being diagnosed with breast cancer during pregnancy	In-depth one-on-one interviews in a public hospital	5 pregnant women Partners: not included	**Clinical** Cancer type: breast cancer Cancer stage: not reported Treatment: chemotherapy Gestation at diagnosis: not reported Point of cancer care: about to start or recently started chemotherapy Parity: 3 multips; 2 primips Conception: 1/5 IVF **Demographics** Age: 38.2 (SD 5.1) Relationship status: married/ co-habiting	All invited patients participated in the interviews 100%	N.A	Qualitative-interpretive phenomenological analysis	Recruitment: not reported Data collection: July 2019–February 2020 (before COVID-19 outbreak)
[16] Italy	To investigate maternal representation in pregnant women with experience of breast cancer and those with no oncological history	Semi-structured interviews in hospital, outpatient study in Lombardy, Italy. Women were in 3^rd^ trimester of pregnancy	**Group 1**—19 women-oncological sample. 4 GBC; 15 previous BC **Group 2**—19 women-non oncological sample with no other medical conditions Partners: not included	**Group 1** **Clinical** Cancer type: breast cancer Cancer stage: not reported Treatment: not reported Gestation at diagnosis: not reported Point of cancer care: not reported Parity: 13 multips; 6 primips **Demographics** Age: 32–41 years Relationship status: heterosexual relationship living with partner of child Employment: GBC 50% employed, 25% education, 25% unemployed; Previous BC 73% employed, 13% education, 7% healthcare professional. 7% unemployed **Group 2** **Clinical** Cancer type: no cancer or medical conditions Cancer stage: N.A Treatment: N.A Gestation at diagnosis: N.A Point of cancer care: N.A Parity: 10 primips, 5 multips **Demographics** Age: 30–41 Relationship status: heterosexual relationship living with partner of child Employment: 63% employee, 16% education, 11% healthcare professional, 10% unemployed	Not reported	N.A	Qualitative thematic approach	Recruitment: not reported Data collection: not reported
[17] Singapore	To explore the experiences of Asian women with gestational breast cancer	Semi-structured, individual, face to face interview in a private room at the breast center of a tertiary women and children’s hospital	7 women with gestational breast cancer (determined by theoretical saturation) Partners: not included	**Clinical** Cancer type: Breast cancer Cancer stage: stage 2–3 Treatment: Surgery and Chemotherapy, Radiation, targeted therapy or hormone therapy Gestation at diagnosis: (1) 1^st^ trimester; (1) 2^nd^ trimester; (1) 3^rd^ trimester and 4 postpartum Point of cancer care: active treatment Parity: 6 multips; 1 primip **Demographics** Age: 21- 40 years Relationship status: all married and co-habiting Employment: 3/7 working	7 participate in the study. Data collection stopped when at participant 7 when data saturation was reached	N.A	Qualitative descriptive study with purposive sampling	Recruitment: Not reported Data collection: Not reported
[18] France	To evaluate how pregnancy and motherhood can influence the mother’s adjustment to cancer	Group 1 and 2—self-administered psychological measures MAC44; Group 1 only semi-structured interviews within French various hospitals	**Group 1-** 24 women -pregnant with cancer **Partners: not reported** **Group 2**—19 non-perinatal women with cancer **Partners: not reported**	**Group 1** **Clinical** Cancer type: breast cancer (75%) or brain, colon, Hodgkin, or melanoma (25%) Cancer stage: not reported Treatment: lumpectomy or mastectomy and chemotherapy radiotherapy, hormone therapy or targeted therapy Gestation at diagnosis: not reported Point of cancer care: 7/24 under treatment; 17/24 remission Parity: 9/24 primips; 15/24 multips **Demographics** Age: 34.6 (28–42 range) Relationship status: 87% married or cohabiting; 13% single Employment: 96% employed **Group 2** **Clinical** Cancer type: breast cancer (non-perinatal, non-metastatic breast cancer, aged less than 45 years) Cancer stage: Treatment: lumpectomy or mastectomy and chemotherapy radiotherapy, hormone therapy or targeted therapy Gestation at diagnosis: not reported Point of cancer care: 17/19 in active treatment Parity: not reported **Demographics** Age: 38.3 (32–44 range) Relationship status: 69% married or cohabiting; 31% single Employment: 95% employed	**Group 1** 29 invited to participate—24 participated **Group 2** 28 invited to participate—19 participated	Group 1—24 women completed self-administered psychological measures MAC44 18/24 women responded to the semi-structured interview	Exploratory comparative mixed method study	Recruitment: 2011 and 2014 Data collection: not reported
[19] Brazil	To investigate how the diagnosis of cancer during pregnancy and the family experience of maternity	2 specialized oncology services in the state of Rio Grande do Sul. Interviews conducted in homes and one conducted via Google Meet	12 women diagnosed with cancer during pregnancy and 19 of their family members (biological, affective or by affinity)	**Clinical** Cancer type: breast cancer, 1 cervical and 1 Hodgkin lymphoma Cancer stage: breast cancer-chemotherapy, radiotherapy and surgery; cervical cancer; hysterectomy; and Hodgkin lymphoma-chemotherapy and radiotherapy Treatment: Gestation at diagnosis: 4 women in 1st half or pregnancy, 5 in 2nd half of pregnancy and 3 postpartum Point of cancer care: 2/12 considered cured; in remission and remained on medication, chemotherapy or radiotherapy Parity: not reported **Demographics** Age: 26–41 years Relationship status: not reported Employment: not reported	Determined by theoretical saturation	Not reported	Qualitative study using theoretical framework-symbolic interactionism	Recruitment: not reported Data collection: March 2018–March 2019
[20] Australia	To explore the health care experiences of women diagnosed with GBC to inform and improve clinical care	Telephone interviews	17 women diagnosed with gestational breast cancer between 2008 and 2013 **Partners:** not included	**Clinical** Cancer type: breast cancer Cancer stage: not reported Treatment: not reported Gestation at diagnosis: 5 first trimester; 4 s trimester; 6 third trimester; 2 postpartum Point of cancer care: not reported Parity: 6 primips; 11 multips **Demographics** Age: 31–43 years (at diagnosis) Relationship status: 100% married/de facto (at diagnosis) Employment: not reported	24 responded, 4 were not eligible and 3 did not respond to consent forms after reminder 17/21 response rate	N.A	Qualitative-semi-structured interview	Recruitment: August 2013 Data collection: August 2013–June 2014
[21] US	To explore variables associated with long-term psychological distress in women following a cancer diagnosed during pregnancy	Robert Wood Johnson Medical School	74 women	**Clinical** Cancer type: breast cancer, Hodgkin’s lymphoma, ovarian, melanoma, other Cancer stage: all stages Treatment: surgery, chemotherapy and/or radiotherapy Gestation at diagnosis: 14.7 weeks (SD 8.4) Point of cancer care: 3.8 years (SD 2.5) since diagnosis Parity: 25 primips; 48 multips **Demographics** Age: 34.2 years at diagnosis Relationship status: not reported Employment: not reported	Not reported	N.A	Quantitative cross-sectional survey	Recruitment: not reported Data collection: not reported
[22] Australia	To explore the psychosocial experiences of pregnancy in women diagnosed with breast cancer during or shortly after pregnancy	Retrospectively identified from a concurrent study dataset in Western Australia	15 women diagnosed with gestational breast cancer **Partners:** not included	**Clinical** Cancer type: breast cancer Cancer stage: not reported Treatment: not reported Gestation at diagnosis: 1 1^st^ trimester; 1 2^nd^ trimester; 2 3^rd^ trimester; 11 postpartum Point of cancer care: retrospective 9–17 years since diagnosis Parity: 3 primips; 12 multips **Demographics** Age: 29–43 years Relationship status: not included Employment: not reported	65 invitations sent, 15 responses. Response rate: 23%	N.A	Qualitative-semi-structured interview	Recruitment: not reported Data collection: August 2005–March 2008
[23] Japan	To clarify the experience of pregnant women with cancer in decision making and to consider the role of nurses in providing care to pregnant women with cancer during their decision-making	Urban university hospital	8 women diagnosed with cancer during pregnancy **Partners:** not included	**Clinical** Cancer type: 3 cervical cancer, 2 breast cancer, 2 leukemia, 1 digestive cancer Cancer stage: not reported Treatment: during pregnancy—breast surgery, chemotherapy, and cervical colonization. After pregnancy—chemotherapy, surgery, and radiotherapy. Gestation at diagnosis: 7 1^st^ trimmest, 1 2^nd^ trimester Point of cancer care: Treatment completed with no reoccurrence Parity: 4 primips; 4 multips **Demographics** Age: 35.5 years Relationship status: not included Employment: not reported	8/15	N.A	Qualitative design Purposive sampling method and retrospective approach	Recruitment: not reported Data collection: June 2011 to January 2012
[24] UK	To explore young women’s experiences of breast cancer	Not reported	3 pregnant women diagnosed with Breast Cancer during pregnancy Partners: not included	**Clinical** Cancer type: breast cancer Cancer stage: not reported Treatment: lumpectomy, chemotherapy and radiotherapy. Gestation at diagnosis: 2 2^nd^ trimester; 1 3^rd^ trimester Point of cancer care: posttreatment Parity: all primiparous women **Demographics** Age: 38, 31, and 27 years Relationship status: not reported Employment: not reported	Not reported	N.A	Qualitative research using grounded theory and feminist methods	Recruitment: not reported Data collection: not reported
[25] Australia	To identify the features enhancing quality healthcare experiences for women with gestational cancer and the impact of the heterogeneous Australian healthcare system	Examination of a subset of data from a larger project–INTEGRATE	23 women diagnosed with gestational cancer (determined by data saturation) **Partners: not included**	**Clinical** Cancer type: 5 hematology; 1 bowel; 15 breast; 1 cervical; 1 lung Cancer stage: not reported Treatment: not reported Gestation at diagnosis: 17.52 SD 10.09 range 0–35 weeks Point of cancer care: not reported Parity: not reported **Demographics** Age: 32.8 SD 3.33 (at diagnosis) Relationship status: 22 married/de facto; 1 separated/ divorced Employment: not reported	23/28; 2 could not be contacted; one withdrew because she wanted distance from her experience; 2 terminations were excluded from analysis	N.A	Qualitative semi-structured interviews	Recruitment: Not reported Data collection: November 2018–May 2019
[26] Belgium and The Netherlands	To identify women and partner at risk for high distress based on coping profile	Questionnaires organized by the International Network on Cancer, Infertility and Pregnancy	61 pregnant women diagnosed with cancer and 61 partners. Total 122 participants	**Clinical** Cancer type: breast cancer; hematological; cervical; ovarian; tongue; Ewing sarcoma and gastrointestinal stromal tumor Cancer stage: stages 1–3 Treatment: surgery only; chemo only; radiotherapy only; surgery & chemo; surgery & radiotherapy; surgery & chemo & radiotherapy; no treatment; and Herceptin Gestation at diagnosis: median gestation 16 weeks Point of cancer care: 70.4% retrospective participants and 29.5% prospective participants. Prospective participants interviewed after treatment decisions had been made Parity: 44.2% primips; 55.7% multips **Demographics** Age: 32 years (range 22–42) Relationship status: not reported Employment: not reported	Not reported	N.A	Quantitative Study–CPQ and CERQ	Recruitment: 2008–2011 Data collection: not reported
[27] Canada	To understand women’s experiences of pregnancy and the postpartum following cancer treatment	Interviews conducted via telephone and one via Zoom version 5.0.2, a secure, online videoconferencing platform, participants accessed call from their home with no else present	10 women **Partners:** not included	**Clinical** Cancer: 3 non-Hodgkin’s lymphoma, 1 leukemia, or 7 breast cancer Cancer stage: Treatment: surgery, chemotherapy, radiation, hormone therapy, and/or Herceptin Gestation at diagnosis: 10 months–5 years before pregnancy Point of cancer care: Not reported Parity: 9 primips; 1 multip **Demographics** Age: 31–40 years Relationship status: all in heterosexual relationship and living with their partner Employment:	14 women recruited to study- literature saturation reached at 10 participants	N.A	Qualitative research, grounded analysis	Recruitment: Not reported Data collection: Not reported

*MACC *mental adjustment to cancer scale, *GBC* gestational breast cancer, *GC* gestational cancer, *BC* breast cancer, *BCDP* breast cancer during pregnancy, *CPQ* Cancer and Pregnancy Questionnaire, *CERQ* Cognitive Emotion Regulation Questionnaire

**Table 3:**
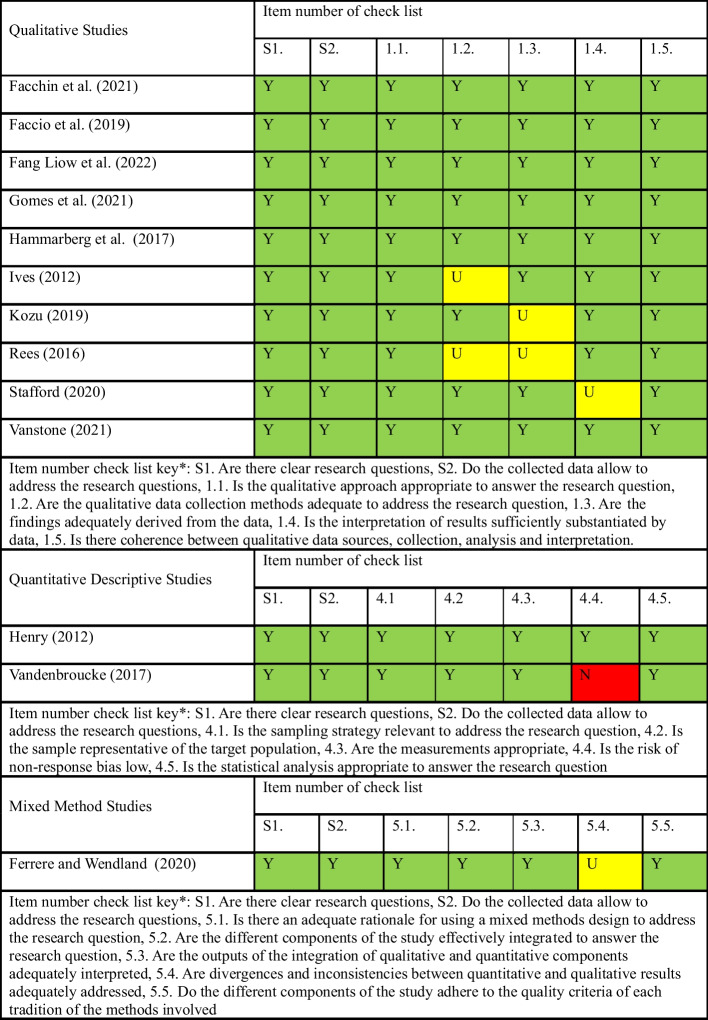
Quality appraisal results

There were 51 individual findings, represented across 13 categories and synthesized into three overall synthesized findings; see Supplementary Table 4. These findings represented the women’s insight into the (1) psychological impact of their gestational cancer, (2) the impact on women’s identity as a mother and a patient, and (3) women’s experiences of complex care. These findings created themes which were further refined into subthemes, outlined in Table 4.

**Table 4:** Themes and subthemes

Themes	Subthemes
Psychological impact	Distress Connections Spirituality
Women’s identity	Femininity Breastfeeding Fertility
Complex care	

### Theme 1: psychological impact

A diagnosis of cancer during pregnancy or the postpartum period had a substantial negative impact on women’s psychological well-being. The impact encompassed women’s expectations, hopes and fears, concerns for their health and wellbeing and that of their baby, the experience of distress, and reflections on spirituality [16, 19, 20, 23–25, 27, 28]

#### Distress

Women experienced distress, which was articulated as feelings of overwhelm, guilt, anxiety, stress, and self-blame which was reflective of their rumination on the conflict of cancer diagnosis and treatment and expectations of pregnancy, birth and mothering [16, 17, 23]. Women reported facing complex, emotional, ethical, and time-sensitive decisions as both a mother and a patient [15, 16, 28].

*“I feel really guilty that I don’t think about what could happen to me, you know, it is still a question mark … also when I have sad thoughts about what I am going through I am afraid that he [the fetus] can feel it.” p 6* [16]

The experience of distress increased significantly in certain circumstances, such as when women conceived spontaneously, they were advised to terminate their pregnancy, they had a cesarean section, had a preterm birth, experienced breastfeeding difficulties, and had cancer reoccurrence or surgery postpregnancy [22]. These experiences were associated with short- and long-term implications as they transitioned through pregnancy, cancer, motherhood with a lasting psychological impact [19, 20, 23]. From the point of diagnosis and throughout their cancer journey, women reported high levels of anxiety and distress. The negative side effects of treatments affected women’s capacity to fulfil their carer role and exacerbated the psychological impact [19, 23]. Long-term implications referenced the aspects of future planning for women and their families. Future planning was focused on fertility and supporting ongoing family life [15, 23, 25]. Women discussed the heavy presence of fear associated with cancer reoccurrence that was balanced against the reflective desire to plan a future family life [19, 20].

*“It was surreal. We are enjoying the pregnancy, telling our friends, and then there’s a bomb like that, it’s as if the ground opened up all at once. How can you have and treat cancer with a baby in your womb? How? We were very anxious and scared!” p4* [20].

#### Connections

Women spoke of their intuitive and tenable connection to their baby, who was consciously considered or prioritized within their care [16, 24, 26]. The innate maternal connection often conflicted with their partner or families, where they often prioritized the care needs of the woman, above the unborn baby, thereby highlighting a supportive care conflict within the experience of gestational cancer among the family unit [24]. Interestingly, when women reflected on their existing children and family, the psychosocial impact turned to practical components including family dynamics, work, and their futures [16]. These insights highlighted the extent of the mental load and burden on women who reported to prioritize needs of her family around her cancer care [16].

*“My family made me think that I have to take on all the treatment… I feel that they need me… They need a Mother… I don’t want them to grow up in an incomplete family, so I have to try harder to actually recover from the illness and win the war” p E266* [17]

The way in which women processed their diagnosis and treatment appeared to be positively influenced by the role of their support persons. The role of partners, family, and friends supported a sense connectivity and determination in their care [17]. Where women and their partners used positive coping mechanisms that included but were not limited to seeking and accepting support, exercise and healthy eating, and joyfulness, there was a notable positive affect [27].

*“He [husband] is always very positive, he cheers me up. He keeps telling me to think positive because things will be alright” p.5* [17].

This finding contrasted with other women who reported an internalized emotional distress which increased their levels of measured distress [26]. When a women’s partners struggled with the diagnosis, women reported that it was hard to cope, which highlighted the importance of partners and familial supports, and the shared experience of gestational cancer.

*“If I cry, he [husband] cries, and this pushes me down. [...] I kept hanging out with friends, going to the gym. I didn’t retreat into myself. I am worried, but I also need distractions” p.5* [17].

#### Spirituality

Women discussed their spirituality through their faith or inspired inner strength [15, 16, 26]. Spiritual reflections were noted to improve women’s psychological wellbeing, which were mostly centered around their own self-determination and positive mindset [27]. This perspective was echoed in both qualitative [15, 16, 21, 25, 27] and quantitative data where women’s “fighting spirit” was noted as a positive psychological adjustment to cancer [19]. Conversely, women shared personal reflections of self-blame, intrusive thoughts, and hyperarousal which negatively impacted their psychological wellbeing [22, 26].

*“I’ve done everything there was to do because I wanted to live for my son, I wanted to stay alive … It was very stressful, but I had good reason to …” p181* [19]

Overwhelming emotion was reported as a reactionary response to the cancer diagnosis [15]. Women stated being shocked by their diagnosis as it arose during their pregnancy or their postpartum period. They envisaged their own mortality and reflected the implications for their family and spiritual aspects of existential distress [15, 23].

*“When they tell you this (the diagnosis), it may initially sound unbelievable, but you immediately see yourself dead, for sure …” p4* [15].

### Theme 2: women’s identity

Women reflected on their “sense of self” while navigating cancer, pregnancy, and postpartum as a mother and a patient which caused a significant shift their identity. Many women discussed their innate femininity, their ideals and expectations of motherhood including birth and breastfeeding, future fertility, and how their sense of self was impacted by their cancer diagnosis.

#### Femininity

Feminine identity was reflected in the women’s own individual ideals of femininity and the perceived value of and changes to her femme attributes such as hair, breasts, weight, birth, ability to breastfeed, and the ability to conceive [15]. Women compared themselves with other women in relation to their health, well-pregnancies, and breastfeeding abilities [15] and reflected on their identity as gestational cancer reshaped their maternal ideal before having cancer.

*“I used to be pretty, but now I have been packing 40 extra pounds and my hair is going to fall out in chunks. He [husband] says: Shave it! I try to hold on, but as I touch my head, a tuft of hair falls out” p.5* [15]

#### Breastfeeding

Breastfeeding had a profound impact on women’s identity which came as a shock to many women, given the context of their diagnosis [28]. Women experienced many breastfeeding challenges, where they were unable to breastfeed their baby due to chemotherapy treatments or breast surgeries. Women were unexpectedly required to reconsider their infant feeding decisions and practices which had a lasting impact on their postnatal experience [23, 28].

*“Breastfeeding, I can’t breastfeed her which I never thought I would actually care about but I have struggled with it. When she was in NICU and the … Lactation consultants would come by and give me a look. Don’t come near me, I can’t breastfeed”. P7* [28].

#### Fertility

Fertility was similarly affected by gestational cancer as women were required to delay conceiving or were unable to conceive [25]. Fertility was affected by treatment, surgery specifically due to hysterectomies and increasing consequences due to age [24, 25]. The option of egg freezing was often not offered or considered and may have contributed to reduced future fertility options for many women [27]. How women reported they felt about their future fertility varied between those, with, or without children, and their hopes and desires for more children. Regardless of the individual situation, fertility concerns affected women’s identity, yet were rarely discussed with the healthcare professional [25].

*“The thing about not being able to have kids as well you know, that’s difficult because that just takes away your, I guess your femininity, just a little bit more” p255* [25].

### Theme 3: complex care

This theme encompassed women’s care experience through their interactions with specialist providers, multidisciplinary care coordination, consultation and referral pathways, decision making, education, and information sharing, and supports [15–17, 21, 23, 24, 27, 28]. Women faced complex decision-making, which was ethically challenging, time-sensitive and long-lasting. The finality of decisions had both short- and long-term impacts, such as profound distressing long-lasting consequences of termination of pregnancy on emotional well-being, preterm births, and neonatal care. Birth plans, breastfeeding, and future fertility were also impacted.

*“It [termination] does make me feel, you know, quite bad some days. It doesn’t go away” p758* [23].

Specialist care was discussed by women as they navigated gestational cancer from diagnosis to treatment. Women mostly reported ongoing communication with their “cancer team” which was inclusive of oncologists, surgeons and cancer nurses, throughout their care. Maternity care was mostly related to the timing of birth with obstetricians and neonatologists, with limited discussion of midwifery care [21, 24].

*“I know that I’d been discussed at both meetings, so the doctors have all spoken to one another and I’ve seen the letters that have been going back and forward between the oncologist and the radiation oncologist, and my GP has been kept in the loop the whole time. And then they’ve also contacted my obstetrician to check when—I think basically the ‘cancer doctor team’, I call them—the cancer team—made decisions that this is what we’d like to do, and then they consulted the obstetrician to make sure that that would work with the baby” p.4* [21]

The experience of care between cancer and maternity care varied significantly. Women reported that their individualized experiences were shaped by healthcare systems, collaborations and entity specifications relevant to both the type of cancer and stage of pregnancy or postpartum [21, 28]. Collaborating care teams demonstrated their own adeptness in supporting women with gestational cancer which was recognizable to the women receiving care. Most notably, women identified gaps within their maternity care experience which was detailed to their cancer diagnosis or treatment outcome.

*“I found that when I was dealing with the cancer- because I was sort of basically dealing with two departments: the maternity department and the cancer department, and when I was in the cancer ward that was fine, can they deal with me- I was a pregnant woman- but it was more dealing with the maternity department. They really struggled to deal with me because I didn’t really fit into any boxes.” p4* [21]

Women revealed they accessed support through partners and family, support groups, and survivors. The uniqueness of their condition was remarked on again as some women struggled to find connections within support groups, while others found opportunities to network with fellow survivors. Most remarkable was the strength that women gained with their personal support network where they found connection and inspiration. [16].

*“But people have been very compassionate. You know, they really dig deep and ask—my emotions; how am I going mentally? You know, physically I can do it all. They’re really concerned about sort of the holistic approach for me, like my whole body, not just, “Well, we’ll just take your boobs off and that’ll be that.” p.6* [21]

## Discussion

Women affected by cancer in pregnancy, and up-to-one-year postpartum, face two transformative and paradoxical life experiences simultaneously. The experience of navigating these significant yet conflicting health events is reported as an isolating experience with one woman aptly reflecting, “I didn’t really fit into any boxes” p. 4 [21]. The findings revealed personalized commentaries on the impact to women’s psychosocial wellbeing and their identity, extending from their sense of self to their breastfeeding expectations and consequential infant feeding alternatives. Women’s experiences were compounded by the complexity of their care and decision-making which was dyadic as it affected the woman and fetus. These findings highlight the unmet supportive care needs that affected women’s psychological, physical, emotional and social supportive care domains thus indicating that research is needed to consider how gestational cancer care is provided and guidelines around models of care.

Women’s wellbeing through pregnancy and cancer is emotionally complex as it ignites reflections of hopes and fears, layered with anxiety, stress, and guilt, all of which contribute to the experience of psychological distress, and reflecting unmet supportive care needs. The unmet supportive care needs of people affected by cancer is well documented within the broader field of cancer care research [29]. Higher levels of distress and anxiety increase subjective care needs [30], a finding that was further reinforced in this study. Supportive care needs, should be individualized to reflect a woman’s needs, by cancer type, gestation, location and relevant demographics so that a suitable multidisciplinary care team is developed [30]. For example, breast cancer reports the most significant unmet needs, owing mostly to burdensome treatments, as it impacts on their physical needs, whereas gynecological cancers impact identity and sense of self [30]. It is important for research to consider how cancer-specific care is proposed within models of gestational cancer care so that entity-specific care is addressed relevant to gestation.

There are other maternal complexities that arise during pregnancy, such as Gestational Diabetes Mellitus (GDM) that display similarities to our findings and are indicative of a wider issue of women’s needs not being met in maternity care provision. A GDM diagnosis requires immediate lifestyle modifications, testing, and treatment protocols. GDM alters the maternal and fetal risk profile, as does cancer, increases the requirements for interventions and impacts short- and long-term health outcomes [31, 32]. Similar to the women in our review, women affected by GDM report significant unmet supportive care needs, [31, 33]. Women diagnosed with GDM describe anxiety, stigma, and disruption within their pregnancy, reinforcing the psychological impact as an unmet supportive care need. Research in this space now suggests that maternity care provision for women diagnosed with GDM be tailored to meet their healthcare needs, with consideration to cultural and racial diversity [34] echoing similar care requirements for those individuals with cancer. Our findings also suggest that cultural considerations were country specific and did not report on individual culture experiences, thus noting a neglect of culture care considerations for women affected by gestational cancer. Research is required to understand cultural needs within gestational cancer and how care can support cultural respect and responsivity.

Women reported overwhelming emotions at the significance of their breastfeeding experience, in synchrony to the absence of discussion or adequate support for breast or infant feeding. The absence of a positive breastfeeding experience raises significant health issues. Breastfeeding is an essential aspect of care for both mother and babies with benefits across their life spans [35]. Women affected by gestational cancer are negatively impacted in their ability to breastfeed owing to the toxic effects of chemotherapy, radiotherapy, targeted therapy, immunotherapy, hormonal therapy drugs and surgeries specifically mastectomies. Many women express a desire to breastfeed and require support to plan breast and infant feeding choice [21, 23, 25]. The stigma associated with breast and infant feeding choices may amplify the perceived experience of women affected by gestational cancer. Further research to explore the experiences of breastfeeding with women affected by gestational cancer and in turn the experience of midwives and lactation consultants providing breastfeeding supports to women is warranted.

Our findings also highlight how women experience a unique isolation, one that is associated with the diagnosis and or treatment of cancer in pregnancy and the postpartum period. Their experiences appear to be further exacerbated by a model of care that is focused on treatment pathways and birth planning, rather than enabling supportive care to enhance wellbeing through the perinatal and cancer continuum. As women affected by gestational cancer are consumers of both cancer care and maternity care services, it is imperative that research and clinical practice integrative their supportive care within proposed models of care. In Australia, women are eight times more likely to switch to a private obstetrician for their maternity care following a diagnosis of cancer in pregnancy [7]; this may reinforce the objective a biomedical care focus on birth planning and mode of birth. While obstetric care is an essential multidisciplinary care component, there is an opportunity to consider how midwifery continuity of care may improve care experiences and address the reported gaps [28].

Midwifery-led continuity of care (CoC) is known to improve satisfaction, participation, and outcomes for women accessing maternity care [36, 37]. CoC practices woman-centered care with a known midwife, who partners with women throughout their antenatal, intrapartum, and postnatal care, completing care, assessments, plans, and referral as appropriate [36]. Typically, CoC is offered in a low risk setting however recent research indicates that satisfaction can be extended into high-risk settings. A recent integrative review notes that women reported better communication, supports and standards of care alongside tertiary obstetric referrals or presentation of high-risk complexities [38]. Women affected by gestational cancer may benefit from midwifery- led CoC to address their supportive care needs. Our findings revealed that women reported significant challenges in breastfeeding and wellbeing [21]. These experiences may have been lessened by the ongoing support of a known midwife working to her full scope of practice, in the presence of a trusting relationship and collaboration within the healthcare team. Research is required to investigate midwifery care with women affected by gestational cancer.

The incidence of gestational cancer is rising in parallel to rising rates of early onset cancers, globally [2, 39]. There is an urgent need to understand the uniqueness of gestational cancer experiences. Effective care navigation that supports both the immediate and long-term health and wellbeing outcomes and experiences for women affected by cancer in pregnancy and up-to-one year postpartum is essential. These findings present significant opportunity for further research to explore women’s experiences of gestational cancer. Research must investigate holistic and integrative supportive care provision with midwives and cancer care nurses who may be suitable to support the identified gaps in psychological, physical, emotional, and social supportive care domains.

### Strengths and limitations

This paper is the first known systematic integrative review to examine the broad range of experiences of women affected by gestational cancer. The thematic analysis of 13 studies from multiple country settings reported overwhelmingly that women’s experiences are affected by the way they receive and navigate care, access supports, and perceive their wellbeing. The findings present significant opportunity for further research to explore maternity care provision, breast and infant feeding, and psychological wellbeing supports.

The limitations of this paper note that all studies in this review were conducted pre-SARS-CoV-2, meaning little is known about the experiences of gestational cancer during the recent pandemic. This paper does not discuss partners or support people’s experience of gestational cancer. Nor does this paper detail the experiences of palliation in gestational cancer care, as current research is focused on survivorship. These identified limitations reflect opportunities for additional research.

It is important to consider that the women represented in this systematic integrative review were mostly survivors of gestational cancer. There was little representation from women who were diagnosed with incurable cancer or experienced disease progression or relapse including experiences of end-of-life care.

The studies included within the paper revealed data from eleven well-resourced country settings, however none of these studies detailed specific cultural considerations or experiences. Future research is required to examine specific cultural care needs of women affected by gestational cancer. Furthermore, as only English language studies were included in the eligibility criteria, some data may not have been incorporated.

## Conclusion

The aim of this systematic integrative review was to understand women’s experiences of gestational cancer during pregnancy and up-to-one-year postpartum. The findings revealed insights into the psychological impact of women’s gestational cancer, the effect on women’s identity as a mother and a patient, and women’s experiences of complex care. Gestational cancer reflects an emerging focus of clinical practice and an opportunity for new research to explore woman-care exploring supportive care needs and models of maternity care.

## Supplementary Information

Below is the link to the electronic supplementary material.

Supplementary file1 (DOCX 64 KB)

## Data Availability

No datasets were generated or analysed during the current study.
