# Supplementary material for: “I didn’t really fit into any boxes”: understanding the experiences of women affected by cancer in pregnancy and up to one-year postpartum—a mixed-method systematic review
Source: J Cancer Surviv. 2024 Oct 26;20(3):895–909. doi: 10.1007/s11764-024-01695-z (PMC13144245; doi:10.1007/s11764-024-01695-z)
Supplement: Supplementary file 1 — Supplementary file1 (DOCX 64 KB) [file 11764_2024_1695_MOESM1_ESM.docx]

**Supplementary tables: “I didn’t really fit into any boxes”: Understanding the experiences of women affected by cancer in pregnancy and up to one-year post-partum. A mixed method review**

**Supplementary Table 1: Search Strategy**

**Search strategy**

Five databases were searched on 7 March 2024 to identify relevant studies. These were APA PsycINFO (via EBSCOhost), CINAHL (via EBSCOhost), Medline (via EBSCOhost), Scopus and Web of Science Core Collection. No limiters were placed on the searches. Searches returned a total of 8,867 results. Search terms and number of results by database:

| **Database** | **Key words** | **Search results** |
| --- | --- | --- |
| **APA PsycINFO** | ((((pregan* OR antenatal OR perinatal OR postnatal OR childbirth OR birth*) AND (cancer* OR neoplasm* OR leuk#emia OR lymphoma OR malignan* OR melanoma OR oncolog* OR tumo#r*)) OR (gestational-trophoblastic-disease)) AND (care OR counsel#ing OR daily-living OR decision* OR emotional OR end-of-life OR faith OR family OR financial OR health-system OR human OR information* OR inter-personal OR need* OR nutrition* OR pain OR palliative OR patient OR person-cent#red OR physical OR physiological OR planning OR psychological OR religious OR social OR spiritual* OR support* OR symptom* OR therap* OR treatment) AND (experience* OR feeling* OR perception* OR perspective* OR view*)) | **442** |
| **CINAHL** | ((((pregan* OR antenatal OR perinatal OR postnatal OR childbirth OR birth*) AND (cancer* OR neoplasm* OR leuk#emia OR lymphoma OR malignan* OR melanoma OR oncolog* OR tumo#r*)) OR (gestational-trophoblastic-disease)) AND (care OR counsel#ing OR daily-living OR decision* OR emotional OR end-of-life OR faith OR family OR financial OR health-system OR human OR information* OR inter-personal OR need* OR nutrition* OR pain OR palliative OR patient OR person-cent#red OR physical OR physiological OR planning OR psychological OR religious OR social OR spiritual* OR support* OR symptom* OR therap* OR treatment) AND (experience* OR feeling* OR perception* OR perspective* OR view*)) | **772** |
| **MEDLINE** | ((((pregan* OR antenatal OR perinatal OR postnatal OR childbirth OR birth*) AND (cancer* OR neoplasm* OR leuk#emia OR lymphoma OR malignan* OR melanoma OR oncolog* OR tumo#r*)) OR (gestational-trophoblastic-disease)) AND (care OR counsel#ing OR daily-living OR decision* OR emotional OR end-of-life OR faith OR family OR financial OR health-system OR human OR information* OR inter-personal OR need* OR nutrition* OR pain OR palliative OR patient OR person-cent#red OR physical OR physiological OR planning OR psychological OR religious OR social OR spiritual* OR support* OR symptom* OR therap* OR treatment) AND (experience* OR feeling* OR perception* OR perspective* OR view*)) | **4,185** |
| **SCOPUS** | ((((pregan* OR antenatal OR perinatal OR postnatal OR childbirth OR birth*) AND (cancer* OR neoplasm* OR leuk#emia OR lymphoma OR malignan* OR melanoma OR oncolog* OR tumo#r*)) OR (gestational-trophoblastic-disease)) AND (care OR counsel#ing OR daily-living OR decision* OR emotional OR end-of-life OR faith OR family OR financial OR health-system OR human OR information* OR inter-personal OR need* OR nutrition* OR pain OR palliative OR patient OR person-cent#red OR physical OR physiological OR planning OR psychological OR religious OR social OR spiritual* OR support* OR symptom* OR therap* OR treatment) AND (experience* OR feeling* OR perception* OR perspective* OR view*)) | **1,945** |
| **Web of Science Core Collection** | ((((pregan* OR antenatal OR perinatal OR postnatal OR childbirth OR birth*) AND (cancer* OR neoplasm* OR leuk#emia OR lymphoma OR malignan* OR melanoma OR oncolog* OR tumo#r*)) OR (gestational-trophoblastic-disease)) AND (care OR counsel#ing OR daily-living OR decision* OR emotional OR end-of-life OR faith OR family OR financial OR health-system OR human OR information* OR inter-personal OR need* OR nutrition* OR pain OR palliative OR patient OR person-cent#red OR physical OR physiological OR planning OR psychological OR religious OR social OR spiritual* OR support* OR symptom* OR therap* OR treatment) AND (experience* OR feeling* OR perception* OR perspective* OR view*)) | **1,523** |

**Supplementary Table 2: Data Extraction**

| Study | | Unequivocal | Credible | Not supported | Finding number |
| --- | --- | --- | --- | --- | --- |
| Facchin (2021) | | | | | |
| **Finding** | **Overwhelming Emotions (Feeling Scared and Vulnerable)** | X |  |  | F1 |
| Illustration | “You have to consider the that things (the treatment) may not go as you wish, which makes me sad for those who remain, including my baby, rather than for myself. My husband… a baby without her mother… I mean he has to work and his parents live far away from us, it would be complicated. My parents, it hurts to see them suffer. I am not that worried about my own pain. My concerns are about my close ones” p4  “When they tell you this (the diagnosis), it may initially sounds unbelievable, but you immediately see yourself dead, for sure…” p4  “I am worried about the consequences of chemotherapy on the baby, but I have been reassured by doctors” p5  “My concern is how to explain to him [the other son] why I am going to lose my hair, which I have already cut. . . We tried to take it as a game. . . we did this together as a family, to avoid traumatizing him, and now we have started to buy nice hats, such that he can get used to this” p4  “I am worried I might not make it. I don’t want to leave my baby” p5 |  |  |  |  |
| **Finding** | **Overwhelming Emotions (Feeling shocked and confused)** | X |  |  | F2 |
| Illustration | “When you find out that you have cancer, you immediately picture yourself in a coffin, not to mention how you feel if you have a baby. Without her, I would probably have reacted differently. But with a baby. . . I am completely focused on her. [. . .] When they tell you this (the diagnosis), it may initially sound unbelievable, but you immediately see yourself dead, for sure” (p4)  “I noticed a lump in my breast while I was breastfeeding my baby. I didn’t give much importance to it, I thought it was related to breastfeeding, but then I noticed enlarged lymph nodes in my armpit. I got checked and in a few days I found out it was cancer” p5 |  |  |  |  |
| **Finding** | **Overwhelming Emotions (Thinking straight and taking action)** | X |  |  | F3 |
| Illustration | “It is tough to carry on a pregnancy under therapy, thinking straight is essential” p5  “Things have to be done, you can’t just stay in bed, you have to move on” p5.  “There is no need to mince words, you just have to undergo therapy, without too much theory” p.5 |  |  |  |  |
| **Finding** | **Sense of difference (Experiencing remarkable physical changes)** | X |  |  | F4 |
| Illustration | “I used to be pretty, but now I have been packing 40 extra pounds and my hair is going to fall out in chunks. He [husband] says: Shave it! I try to hold on, but as I touch my head, a tuft of hair falls out” p.5 |  |  |  |  |
| **Finding** | **Sense of difference (Comparisons with healthy women)** | X |  |  | F5 |
| Illustration | “I don’t want to isolate myself because I’m going to lose my hair. I know it might have a strong emotional impact on me… it might cause inhibition…” p6  “… I see other pregnant women and they are more active than me … I’m sorry about that…” p6  “This was supposed to be a serene time for me as for the other women” p.5 |  |  |  |  |
| **Finding** | **Source of strength (being positive)** | X |  |  | F6 |
| Illustration | “Being a smiling person, being able to focus on other things, and thinking positive, despite everything” p6  “I just found out that I can be strong. . . I thought I was weaker. So, I want to be positive, because I noticed that after a few tragic days, I was able to react to the situation” p.5 |  |  |  |  |
| **Finding** | **Source of Strength (Feeling supported and listened to)** | X |  |  | F7 |
| Illustration | “My mom is very positive, too. She tells me to stay calm because I will be fine” p5  “He [husband] is always very positive, he cheers me up. He keeps telling me to think positive because things will be alright” p.5  “My doctor called me and said: ‘It’s been a bombshell, but don’t worry. We have to take care of you and the baby, now. Everything will be fine, you are a lioness’” p.5  “If I cry, he [husband] cries, and this pushes me down. [. . .] I kept hanging out with friends, going to the gym. I didn’t retreat into myself. I am worried, but I also need distractions” p.5 |  |  |  |  |
| **Finding** | **Source of strength (Religion and spirituality)** | X |  |  | F8 |
| Illustration | “I have always been scared by this disease, in the place where I live cases of cancer occur in almost all families, and I have always thought that maybe, one day, that could be me” p.5  “This is God’s will and I’m sure everything will be alright” p6  “I heard that breast cancer, from a spiritual perspective, may be related to something unresolved in one’s history” p.5 |  |  |  |  |
| Faccio (2019) | | | | | |
| **Finding** | **Fears and worries** | X |  |  | F9 |
|  | “I am quite scared about giving birth as I am doing chemotherapy… I really hope that my child doesn’t have any problems as she is living all this with me, I really hope she will be ok” p5  “I am quite worried when I think about breastfeeding, if it will be possible to and if I should breastfeed or not. Breastfeeding would mean waiting, even if it is sort of natural hormone therapy on the other hand it means postponing conventional therapy. So in my case, even if breastfeeding could be beneficial to reduce risk reoccurrence, it means delaying hormone therapy”. P5 |  |  |  |  |
| **Finding** | **Meaning of motherhood** | X |  |  | F10 |
|  | “I was astonished because, honestly, I was not expecting it… but being pregnant is worth everything , it has cast a light in my life, it’s a gift and I think that this gift can help me” p6 |  |  |  |  |
| **Finding** | **Mother-foetus relationships** | X |  |  | F11 |
|  | “I feel really guilt that I don’t think about what could happen to me, you know, it is still a question mark… also when I have sad thoughts about what I am going through I am afraid that he can feel it” p6 |  |  |  |  |
| **Finding** | **Partner role** | X |  |  | F12 |
|  | “I must say that the pregnancy really strengthened us and it’s a moment that pulls you together and strengthened your relationship, in spite of the illness” p7 |  |  |  |  |
| Fang Liow (2022) | | | | | |
| **Finding** | **Being a sick woman** | X |  |  | F13 |
| Illustration | “I’m bald, so I have to wear a hat, but when you step out into society, people look at you in a weird form… You have no fringe, no side burns and no hair so they can predict that you are sick…” p E265  “I thought that it's just a lump…because it's pregnancy, so I thought it's because of the breast milk…so I was thinking that after giving birth, then I go to through the check-up.”E265  “I told myself that I must give myself a short period to allow myself to be sad. To allow myself to have the bad emotions…. After that, I need to be strong…. I cannot just be thinking about the bad things” E267 |  |  |  |  |
| **Finding** | **Juggling between being a Mother and a patient** | X |  |  | F14 |
| Illustration | “A part of me is missing because it’s not as much interaction with my first kid. The second is totally difference whereby I don’t have much playing time with him… so he started to get closer with his Grandma… So it’s like I’m not an important person in his view. So yeah it’s a bit sad to see that” p E266  “My family made me think that I have to take on all the treatment… I feel that they need me… They need a Mother… I don’t want them to grow up in an incomplete family, so I have to try harder to actually recover from the illness and win the war” p E266  “My husband! Yeah, he was there for me. All. Throughout. From my diagnosis up to the end of my treatment. Yeah, he stayed with me… My hair fell…so…I took the courage to go cut botak (Malay word for bald). So, my husband also did the same, so we both cut together….” E265 |  |  |  |  |
| **Finding** | **Seeking normalcy** | X |  |  | F15 |
| Illustration | “Treat me like your friend or your sister or your relative close to you… like a normal person Don’t treat me like a patient…” pE266  “I was reading this article about those breast cancer survivors. I have learned a lot from them… They share their everyday experiences… It helps… I found another support from Them” E266.  “Doctors are professional; we are not the doctors…. We don't know much about this. Even if you can go to Google or something like that, that's not 100% real” E266 |  |  |  |  |
| **Ferrere (2020)** | | | | | |
| **Finding** | **Defense mechanisms to adjust to the disease** | X |  |  | F16 |
| Illustration | “After the announcement, I entered a kind of infernal whirlwind, I totally ignored my pregnancy because death replaced life” p180 |  |  |  |  |
| **Finding** | **Fighting spirit** | X |  |  | F17 |
| Illustration | “I’ve done everything there was to do because I wanted to live for my son, I wanted to stay alive… It was very stressful, but I had good reason on…” p181 |  |  |  |  |
| **Finding** | **Visibility and acceptability of the disease** | X |  |  | F18 |
| Illustration | “At the end of the treatment I was lost, it was all nothingness, it was the beginning of a moral descent, I didn’t feel surrounded, supported anymore, nor cared for and I missed the oncology center. The change from “pregnant” to “ill-pregnant” was difficult and then from “pregnant and sick” to the status of just “sick” was difficult but now I feel nothing, neither pregnant nor sick, I just feel empty” p182 |  |  |  |  |
| **Gomes (2021)** | | | | | |
| **Finding** | **Being surprised by the diagnosis of cancer during pregnancy** | X |  |  | F19 |
| Illustration | “I felt a lump in my breast, but I thought it was milk because it hadn’t been too long since I weaned by second child. I spoke to my mother and she also thought it was that too. That’s is why it took me a while to show it to the doctor” p3  “It was surreal. We are enjoying the pregnancy, telling our friends, and then there’s a bomb like that, it’s as if the ground opened up all at once. How can you have and treat cancer with a baby in your womb? How? We were very anxious and scared!” p4  “The first thing that comes to mind is death. What about the baby? How is it going to be. If I could change all that, but I can’t, it’s one day at a time, not knowing how the next day will be like. From an immense joy to a deep fear of everything” p4  “The postoperative period was very painful, I had a lot of pain and could not take medication because I was pregnant. Everyone cried; it was very difficult for everyone here at home” p3  “You know how it is: she went straight to Google and there’s nothing but bad news there. I said to stop doing that and we started to ask everything to the doctor and believe in what he told us.” p4  “I looked for help for me and my mother. We both went to the psychologist. And the nutritionist also helped me because I could not eat.” p4  “It was her faith that made us believe that everything would work out fine. Because at the beginning I didn’t have hope. She helped us to have faith and believe that it would pass.” p4 |  |  |  |  |
| **Finding** | **Suffering the repercussions of cancer on pregnancy and birth** | X |  |  | F20 |
| Illustration | “It’s like being on a seesaw: the pregnancy and the baby on one side and the cancer on the other” p5  “It’s happiness for the baby, sadness for the disease, and fear for both”. p5  “I can say that I didn’t experience the pregnancy, because I was being examined all the time, chemotherapy, surgery, and then the baby was born. You know that thing of enjoying pregnancy? We didn’t have that. It was different, very different from what we thought it would be.” p5  “What made me suffer the most wasn’t the treatment, it was not being able to breastfeed. I felt incomplete, that I was an incomplete mother, who could not even give the best food to her child. It took me a while to accept this, in fact I think I haven’t’ accepted it until today” p5  “I was very mad when I heard from the doctor that I would have to have an abortion. Never! I will fight for my life and for my daughter’s life. If we got in this together, we will leave together” p5  “I was very confused. I did think about removing the uterus and ending this once and for all. I won’t deny it, I didn’t want to die. My husband wanted to wait. It was a very difficult decision. In fact, I think we never really decided. We just rolled with it.” p5  “It was one day at a time, always with that feeling that we could lose the baby at any time. After chemotherapy, my wife always asked for an ultrasound to see how the baby was doing. This calmed her down” p.5  “That’s exactly what she said: first you couldn’t take medication for your headache, and now they were talking about chemotherapy? During pregnancy? We couldn’t understand how that would be possible. We even talked about postponing it until after the birth, but then we were afraid that it could be too late.” p.5 |  |  |  |  |
| **Hammarberg (2017)** | | | | | |
| **Finding** | **Communication (interdisciplinary communication)** | X |  |  | F21 |
| **Illustration** | “I know that I’d been discussed at both meetings, so the doctors have all spoken to one another and I’ve seen the letters that have been going back and forward between the oncologist and the radiation oncologist, and my GP has been kept in the loop the whole time. And then they’ve also contacted my obstetrician to check when—I think basically the ‘cancer doctor team’, I call them—the cancer team—made decisions that this is what we’d like to do, and then they consulted the obstetrician to make sure that that would work with the baby” p.4  “I found that when I was dealing with the cancer- because I was sort of basically dealing with two departments: the maternity department and the cancer department, and when I was in the cancer ward that was fine, can they deal with me- I was a pregnant woman- but it was more dealing with the maternity department. They really struggled to deal with me because I didn’t really fit into any boxes. You know, here I was, this pregnant woman, but I had cancer.. I just felt they didn’t quite know how to deal with me. And also, you know, those two departments talking to each other about me, I feel they struggled with that as well” p4  “Trying to work out, and getting a bit of mixed opinions on what’s the best course—well especially when I was first diagnosed—the obstetrician thought, “Well you should absolutely have a mastectomy,” whereas the surgeon was  saying, “Well I think a mastectomy is a bit of overkill considering the size of the tumor that you have.” |  |  |  |  |
| **Flinding** | **Communication (patient communication)** | X |  |  | F22 |
| **Illustration** | “So she’d (the GP) sort of done all this prep work in the background before I’d come to see her, which was really good, because when I got there- you know, you’d get the diagnosis, and that’s terrible at the time, but she actually could say, “From here is what’s going to happen”” p5  “I did have the lactation specialist and the midwives and everyone looking at it, and they just said that I had fibrous tissue, and nothing to worry about. So me being a first-time mum had no idea, so I went home. I obviously couldn’t feed out of that breast at all—I said “I’ve had a few problems feeding with one breast,” and then a few people said to me, “You should get it checked out.” And my obstetrician said, “No no, it’s fine,” and I thought, “No, I’ll just go along.” So obviously I went and had a mammogram, and yeah, I had a very large tumor behind my nipple.” p.5  “She [surgeon] spent as much time with me as I needed that day, which is exactly what I needed. I wanted to know the ins and outs of everything, you know…. I just needed knowledge. I just needed information, I think, at that initial time, and to be able to sit down with her was absolutely invaluable” p.5  “The oncologist didn’t tell me a great deal of information about what was going to happen to me. They sort of give you that list of everything that can possibly go wrong. And you walk away thinking, “I wish I hadn’t read any of that.” p.5  “So the doctor that we spoke to, her only—only—suggestion was for me to terminate, and then follow the treatment from there. …We decided to stick with the pregnancy. There was no way I could have gone through a termination—not a hope in Henry! I just couldn’t bring myself to do it.” p.5  “I had a key person that I could contact any time, and that I could ask information, because when I needed to know things or wanted to understand something a bit  better, I could ring her or talk to her. She was very skilled, very knowledgeable. So that made a huge difference.” p.5  “And I didn’t even know that breast care nurses existed until after I’d had my second-last chemo treatment. So I’d been for surgery; I’d been through five chemo treatments; I’d been through hell, pretty much, before I even knew the breast care nurses existed” p.6 |  |  |  |  |
| **Finding** | **Comprehensive care (fighting spirit)** | X |  |  | F23 |
| Illustration | “But people have been very compassionate. You know, they really dig deep and ask—my emotions; how am I going mentally? You know, physically I can do it all. They’re really concerned about sort of the holistic approach for me, like my whole body, not just, “Well, we’ll just take your boobs off and that’ll be that.” p.6  “I sort of felt like she [the counsellor] was trying to prepare me for the worst, and that made me feel really uncomfortable. She gave me some good visualisation techniques and things like that. But yeah, every time I went there, she was like. “And how would you feel if it—”; “How do you feel about death?” and things like that. “Back off, I’m not dying, you people.” So yeah, I stopped speaking to her” p.6  “The best thing that he [the obstetrician] could do for me was to arrange a meeting with the girl that he had treated 5 years earlier that had been through it, and from there we were able to strike up a relationship that, whenever I had a question, I would just text her—doesn’t matter what time of day—and she would get straight back to me and tell me this, that and the other.” p.6  “And I am also in a support group and in that support group there’s three or four women who’ve been through the same thing, so I think that is very important tool, to be able to speak to other women who’ve gone through it and see those kids- to see those children with your own eyes, that everything is ok” p6  “I mean having breast cancer is scary; having breast cancer as a young woman is, you know, scary on top of that and then having a brand new baby. I just felt like there was noone else in my situation, like no-one I could talk to about that. And I went to a couple of support groups and things like that, and I just felt really out of place, and didn’t really feel like they understood exactly” p.6 |  |  |  |  |
| **Finding** | **Comprehensive care (the mind)** | X |  |  | F24 |
| **Illustration** | “He said, “We don’t have any real facts and figures to give you about how the baby will be.” He said, “She’ll be taken out early, and she will be smaller than the normal,” but that’s all they could really tell me, so that was the  frightening part, obviously” p.6  “They offered it [chemotherapy] and said that I could do it while I was pregnant, but there were very big ‘ifs’ and ‘buts’ about what effect it would have on the child. It was ‘maybe this’ and ‘maybe that’ and I just couldn’t—I wouldn’t put my own body through that, so to do that to an unborn child, I couldn’t fathom” p.6  “That’s been really tough. I’m getting a lot of mixed opinions about breast feeding. I breast fed my son for 9 months, and I really enjoyed it, and I was really keen to do it, but I’m getting really mixed messages about whether it will be possible, for how long it would be possible” p.7  “And even though it’s five and a half years ago, I still remember vividly the doctors giving me some tablets to dry up my milk. And that was the hardest—I think that was the hardest part of everything” p.7  “So that [breastfeeding] was out of the question, so I made an appointment to see my maternal health nurse from my first baby. And I went to see her, and she kind  of just guided us in what the best options were with formula feeding, just so we could get our head around it, and just know that that was perfectly good for our baby, and I didn’t need to listen to anyone else” p.7  “I mean, I also found my oncologist wasn’t very forthcoming with information either. He was—again, I think my age had a lot to do with that. I wasn’t his general, you know 60 or 70-year-old lady who had breast cancer, because I had a  lot of fertility questions to ask him—you know, “Can I have babies after this? How’s it going to affect—?” and all he said to me, “Here’s a site; google it; check out your chance of fertility after it.” P.7 |  |  |  |  |
| **Finding** | **Comprehensive care (the body)** | X |  |  | F25 |
| Illustration | “No-one will talk to you about complementary therapies or all those things. I guess I just wasn’t surprised. I mean, I don’t really understand why they don’t sort of—  you know, “This is a really great diet to do,” or suggesting more exercise, or acupuncture. You know, I’m not talking about whacky things, just things that often can help. And yeah, you don’t really get much advice on that. I guess I just sought that out myself” p.7  “The only other criticism I would have is, I had a lot of problems with intimacy and things afterwards because of the hormone treatment, and there’s no real follow-up, or no one to turn to. I found it quite difficult” p.7 |  |  |  |  |
| **Henry (2012)** | | | | | |
| **Finding** | **Distress** | X |  |  | F49 |
| Illustration | Women were more likely to experience long-term distress if  1. they conceived without fertility assistance [adjusted β0.26, p 0.02]  2. had been advised to terminate their pregnancy [adjusted β0.27, p 0.02]  3. had undergone a caesarean section [adjusted β0.23, p0.06]  4. had a preterm baby [adjusted β0.34, p0.005]  5. had not produced enough milk to feed their baby [adjusted β0.29, p0.02]  6. currently experiencing cancer reoccurrence [adjusted β0.26, p0.03]  7. had undergone surgery post-pregnancy [adjusted β0.28, p0.01]. |  |  |  |  |
| **Ives (2012)** | | | | | |
| **Finding** | **Motherhood and the conflict of GBC diagnosis during pregnancy** | X |  |  | F26 |
| Illustration | “I had two young children and I just didn’t want to die, and that was my first thought” p756  “If I had to have a termination, if they said I have to have one, if they said the best option is to have one, I think I would have had one. But then when you come down to it, I didn’t want to because I just, I knew it would have been really, really hard but I’ve got (x) other kids, I can’t sort of risk my life” p756  “Well I wanted, well obviously I wanted the baby but my health had to come first and anyway, (the) biggest thing, and I guess the other disappointment and I suppose there’s nothing I can do about it. Was the age that I was when I got it and then to be told I couldn’t have any, couldn’t have another child for two years.” p756 |  |  |  |  |
| **Finding** | **Termination of pregnancy** | X |  |  | F27 |
| Illustration | “It does make me feel, you know, quite bad some days. It doesn’t go away” p758 |  |  |  |  |
| **Finding** | **Breast cancer treatment during pregnancy** | X |  |  | F28 |
| Illustration | “All I wanted was a healthy baby, and then when he came and said we are delivering him tomorrow I thought thank goodness, I get to see my baby. And I hope he’s going to be ok”  “I was sent in for tests and I had a bone scan and they had to give me the radioactive stuff in nuclear medicine and I was concerned. And they assured me that it would have no effect on the baby or a very minimal effect. I said, ‘I don’t want him to be sterile when he grows up and stuff’. They said, ‘no, no, no. He should be fine’. So that was my main concern was because of that radioactive needle I had to have” p758 |  |  |  |  |
| **Finding** | **Birth soon after a diagnosis of breast cancer** | X |  |  | F29 |
|  | “I remember the following week it was sort of a tug of war, because they called in a specialist obstetrician as well. And he wanted the baby to stay in utero as long as possible (37 weeks gestation)… and they called in the oncologist and he wanted the baby delivered quickly because he wanted to start my chemo(therapy)… They were both coming in and have consultations, and my obstetrician would be going “tut, tut, tut,… this baby is not ready. I’m not delivering” And I’d be taken down for ultra sounds t check lung movement and stuff. Oh it was beautiful to see the baby on the ultrasound and he seemed unscathed, and they assured me he would be fine and all of it… it was quite a relief down the track” p758 |  |  |  |  |
| **Finding** | **Post delivery** | X |  |  | F30 |
| Illustration | “I cried when he was born, I never cried with any of my other kids. I think I was just glad that he was out and away from the chemotherapy and everything. And then I cried again when I saw him because he was in like the little crib and all the tubes coming out of him and everything and I felt very bad because I’d made him be in there because I’d chosen to have him” p758 |  |  |  |  |
| **Finding** | **Experiences of breast and bottle feeding** | X |  |  | F31 |
| Illustration | “After I’d had the baby and um, I didn’t feel he was getting enough milk off one breast, and I’d been told like try and change the position. So feed him on that side, then turn him around to this side and um have another go at feeding him. But then my nipple was getting so sore. So I went down to the nursery and I sat in the chair to feed and I asked one of the midwives something about the feeding, and she said oh yes dear, but um. You only feed for ten minutes on that side and then ten minutes on the other side. And I said oh, I’m sorry but I’ve had the mastectomy. And she said oh, oh, you’re that lady. Oh sorry. And she was so apologetic. And she was going I’m sorry, I’m sorry, I’m so sorry. I’m like that’s OK and then I took the baby and just went back to my room. So it was like, um I didn’t belong in the maternity ward.” p759  “I think I was concerned about being able to continue breastfeeding but ultimately I thought it was probably better for me to just be alive. A formula fed baby will be fine and better to have a mother than not.” p759 |  |  |  |  |
| **Kozu (2019)** | | | | | |
| **Finding** | **Interaction between the woman and her medical team, fetus, family members and medical staff** | X |  |  | F32 |
| Illustration | “My head was full of cancer rather than consider whether I continued or not my pregnancy” p5  “My husband and my best friend told me to prioritize my healing rather than the baby’s life” p6 |  |  |  |  |
| **Finding** | **Confrontation with dilemma and uncertainty** | X |  |  | F33 |
| Illustration | “If I disagree with premature birth, it means a delay in cancer treatment, I would fall ill, I can’t take care of my child. The mere thought of it troubles me deeply. This decision-making was quite difficult for me” p7  “The most anxious and painful thing involved what would happen if I could not give birth” p7 |  |  |  |  |
| **Finding** | **Redefinition of the women’s own decision** | X |  |  | F34 |
| Illustration | “The doctor explained that cesarean section and radical hysterectomy will be performed at the same time next week, and I felt that there was no choice” p8 |  |  |  |  |
| **Rees (2016)** | | | | | |
| **Finding** | **Disturbed expectations** | X |  |  | F35 |
| Illustration | “It was like someone had just said: ‘Did you know you’re having a baby tomorrow?’” p254  “It was what should have been one of the happiest times of my life but it is tainted with one of the worst thing you could ever be told” p254 |  |  |  |  |
| **Finding** | **Implications for motherhood** | X |  |  | F36 |
| Illustration | “”Everybody else y’know at the NTC (National Childbirth Trust) Group continues to breastfeed for six months or even longer” p254  “I could never really allow myself to be that poorly really because, um, because I had to be okay to look after her really. Um, and y’know she’s still here, which is proof that I did an okay job I guess!’ p255  “There’s nothing wrong with [child], you wouldn’t know she was prem or had been through all that strife.” P2565 |  |  |  |  |
| **Finding** | **Future fertility** | X |  |  | F37 |
| Illustration | “It was really hard (but) it was likely that I wouldn’t be able to have more children afterwards so I kind of still wanted to do that, do most of the things for yourself really” p255  “The thing about not being able to have kids as well you know, that’s difficult because that just takes away your, I guess your femininity, just a little bit more” p255  “We’ll try probably in about another year…When you’re told that there’s a possibility you’re not going to be able to have that it makes you want it even more.” P255 |  |  |  |  |
| **Finding** | **Sense of pride** | X |  |  | F38 |
| Illustration | “I just almost want to stand as a bit of symbol maybe like to be really strong and that you can do it, and the fact that I did it with a tiny baby which for most people they struggle anyway with that, being a mum for the first time, I went through all of that on top you just think ’I did it so you can do it’” p256 |  |  |  |  |
| **Stafford (2020)** | | | | | |
| **Finding** | **Control over healthcare** | X |  |  | F39 |
| Illustration | “I had two kids and I’m the full time carer at home, I needed to be with a schedule, when I would be able to have those treatments and the time.. I couldn’t wait to be told, I needed to be able to schedule it myself. And I was given assurances. So I was going to have that flexibility” p5  'I was thinking about alternative medicine as well to coincide with western medicine. And would it be okay? Were there any journal articles to suggest something like acupuncture …And did he know anything about that… every question I asked him he went and researched. And then forwarded a whole lot of e-mails that night of journal articles of studies that he'd found… He let me do that and so I felt empowered, but it was also my decision.' p5 |  |  |  |  |
| **Finding** | **Trust in clinicians, the hospitals and systems** | X |  |  | F40 |
| Illustration | “'I have complete trust in my obstetrician… she told me straight off all of the information. And she said, if you need the chemotherapy in your second trimester, that it will be okay… She rings up people from… other hospitals… I have faith in her because she delivered [existing child]…I know that she knows the way I am. And so I've just been guided by her and trusted that the information that she's giving me'” p5  “And the GP confirmed it was (cancer). He gave me a referral straight away to the local oncologist. I got home and realized that I don’t really trust the medical system (regional area)… So I turned around and went straight back to the doctors and asked him to give me a referral to a (hospital site in a metropolitan area)… I thought, there’s more chance that they’ve dealt with pregnancy and cancer”p5 |  |  |  |  |
| **Finding** | **Co-ordination of care** | X |  |  | F41 |
| Illustration | “'Working between the [private hospital site] and the public… you couldn't tell they hadn't been doing it for fifty years. They were just so in sync with how they got all of this information across…I never had to repeat myself, and it just made it so much easier to deal with. Yes, it was really good'.”p6  “I felt that was a really important meeting… And I turned up to the appointment, nervous and anxious and worried. And there was no oncology results. Because they hadn’t sent them across… It’s been a little infuriating… when information goes missing or isn’t at hand or scans just hadn’t turned up, it’s not a reassuring feeling at all. I had a joke, before I went into surgery like what if they do the wrong breast” p6 |  |  |  |  |
| **Finding** | **Uncommon diagnosis** | X |  |  | F42 |
| Illustration | “And I think when I was pregnant.. I got the highest standard of care because everyone was so worried about you, your pregnancy everything like and it’s a unique case. And I think that’s why I got such a high standard of care” p7  “'There were NICU, people from upstairs… IVF people… obstetricians… radiologist… the oncologist… nurses… an anesthetist… administrative staff… It was a bit insane… at one point, the anesthetist asked everybody to leave, because it was too noisy… I felt like a little bit of a rock star, like a really special case…and then of course trying to squeeze my husband in next to me … it was a bit of a circus. I didn't want to be that circus act, but there I was'.”p7 |  |  |  |  |
| **Finding** | **Holistic, future orientated care (psychological needs)** | X |  |  | F43 |
| Illustration | “They treated the cancer, the thing to get out, your mental health is a luxury… I found that they treat the cancer not the patient.. you don’t really budget for cancer, how expensive everything is. And so really to then put on top of that to go and see a psychologist… it feels like a luxury… At the end of the week, there not really much left over for you to go and spend $250 on a psychologist appointment” p7  “Breastfeeding, I can’t breast feed her which I never thought I would actually care about but I have struggled with it. When she was NICU and the …. Lactation consultants would come by and give me a look. Don’t come near me, I can’t breastfeed”. p7  “The door's always open. You can come to me any time and just let me know if you want to see someone”. I was given a mental health plan right from the first day and then, my oncologist… said… let me know if you want to see the psychologist here at the hospital. We can arrange it. We can do it now, to see her'. p7 |  |  |  |  |
| **Finding** | **Holistic, future orientated care (Parenting needs and planning)** | X |  |  | F44 |
|  | “'It's horrible. I'm not going to lie. You know exactly why you're having it [radiation therapy], but that doesn't make it any easier to be walking out of that door and leaving my baby… to leave her and go and sit in a hospital and then worry about germs as well. Before she'd had her vaccinations and things that was difficult'” p7  “Breast feeding, I can't breast feed her which I never thought I would actually care about but I have struggled with it. When she was in the NICU a and the… Lactation consultants would come by and give me a look. Don't come near me, I can't breastfeed'” p7 |  |  |  |  |
| **Vanstone (2021)** | | | | | |
| **Finding** | **Preservation of hope** | X |  |  | F45 |
| Illustration | “I was I think grieving this possibility of [infertility]… That was harder for me. I was sad that maybe we won’t be able to have kids, or we won’t for five years or you know, we don’t know if it will even be possible…”p6  “I guess there’s so many things going on at the time of diagnosis, like coordinating a surgeon and oncologist…that maybe it doesn’t seem like as high of a priority, or it gets forgotten because of that” p6  “I feel grateful that I had the opportunity to end up freezing some eggs. I didn’t end up using them but it was actually quite helpful for me in terms of coping…to know that cancer wasn’t taking that away from me, you know, it was still an option.” p7 |  |  |  |  |
| **Finding** | **Joy shaded by worry** | X |  |  | F46 |
| Illustration | “I remember on the hospital tour actually just bursting out - I had to leave and was just in tears, I was like ‘I can’t do this I don’t want to have a hospital birth’... they’re [hospitals] filled with fear”p7  “You do carry with you, kind of like medical trauma…you kind of get into a mindset that you’re always going to receive terrible medical news” p7  “You’re so overjoyed about this baby, but then you’re terrified that your cancer is going to come back” p7 |  |  |  |  |
| **Finding** | **Conceiving of a new future** | X |  |  | F47 |
| Illustration | “A lot of the information… out there were for women who had cancer later on in life after they’ve had kids…That’s all the information I could find” p8  “I remember asking about…chemo and how that would affect fertility and him saying, ‘You know, I don’t really know’” p8  “It (medical system) doesn’t have all the answers, so you do need to be your own advocate” |  |  |  |  |
| **Finding** | **Shedding my cancer body** | X |  |  | F48 |
| Illustration | “Remembering when I thought I couldn’t have kids or just remembering really difficult aspects of going through the chemo…I would say that I do feel almost more distanced from it now that I’m pregnant and going through this…. chapter… I will view being pregnant as the ultimate symbol of being done with it.” P9 |  |  |  |  |
| **Finding** | **Distress** | X | |  | F50 |
| Illustration | Women more inclined to maintain pregnancy than partners. (P=0.011)  32.8% used internalized coping strategies  18.9% blamed themselves or others |  |  |  |  |
| **Finding** | **Cognitive emotion regulation strategies** | X |  |  | F51 |
| Illustration | Participants using internalized emotional regulation had significantly higher distress, specifically concerns about the child’s health (P=.039), disease and treatment (P< .001), and pregnancy and delivery (P= .009) than those using positive coping strategies  48.3% women and partners used positive coping strategies |  |  |  |  |

Definitions of abbreviated terms: NICU - Neonatal Intensive Care Unit

**Supplementary Table 3: Excluded Full Papers**

| **Author and year** | **Title** |  |
| --- | --- | --- |
| Lacour 2005 | Management of cervical adenocarcinoma in situ during pregnancy | Wrong outcome |
| Okujima 2022 | A Case of Breast Cancer during Pregnancy Treated with Neoadjuvant Chemotherapy during Pregnancy | Non English language |
| Pinnix 2016 | Maternal and Fetal Outcomes After Therapy for Hodgkin or Non-Hodgkin Lymphoma Diagnosed During Pregnancy | Wrong outcome |
| Rojo-Contreras 2015 | Clinical evaluation of patients with breast cancer and pregnancy | Non English language |
| Sutton 1990 | Pregnancy and offspring after adjuvant chemotherapy in breast cancer patients | Wrong outcome |
| Smorti 2021 | Protect, promote and support: A warm chain of breastfeeding for oncological women-results from a survey of young Italian cancer mothers | Wrong patient population |
| Greiber 2022 | Cancer in pregnancy and the risk of adverse pregnancy and neonatal outcomes: A nationwide cohort study | Wrong outcome |
| Li 2011 | Neoadjuvant chemotherapy with paclitaxel plus platinum for invasive cervical cancer in pregnancy: two case report and literature review | Wrong outcome |
| Alder 2009 | Psycho-oncological care for young women facing cancer and pregnancy | Non English language |
| Dur 2021 | Impact of pregnancy on cancer survival: Experience at a tertiary care hospital | Wrong outcomes |
| Basu 2011 | A pregnant woman with metastatic papillary thyroid carcinoma and paraplegia: Multiple considerations involved in the management | Publication type |
| AlıcıDavutoğlu 2017 | Pregnancy in cancer patients and survivors; experience of a university hospital in Turkey | Wrong outcomes |
| AzulayChertok 2020 | Infant feeding among women with a history of breast cancer | Wrong patient population |
| Harris 1990 | Issues in nursing care of pregnant patients with cancer | Publication type |
| Safi 2023 | Pregnancy associated cancer, timing of birth and clinical decision making-a NSW data linkage study | Wrong outcomes |
| Melik-Andreasyan 2022 | ABCL-263 Our Experience in the Management of Patients With Lymphoma During Pregnancy | Publication type |
| Antonio 2010 | A group intervention model for women diagnosed with cancer during the prenatal or postpartum period | Publication type |
| Çömez 2016 | We as Spouses Have Experienced a Real Disaster!: A Qualitative Study of Women With Breast Cancer and Their Spouses | Wrong patient population |
| Leung 2020 | Psychological aspects of gestational cancer: A systematic review | Review |
| Sheng 2016 | Successful pregnancy and delivery in a patient with chronic myeloid leukemia: a case report and review of the literature | Wrong outcomes |
| Yu 2013 | Management of ovarian tumors complicated by pregnancy and its influence on pregnancy outcome | Non English language |
| He 2012 | Successful management of mucinous ovarian cancer by conservative surgery in week 6 of pregnancy: case report and literature review | Wrong outcomes |
| Lee 2019 | Metastatic colorectal cancer during pregnancy: A tertiary center experience and review of the literature | Wrong outcomes |
| Bell 2013 | Pregnancy-associated breast cancer and pregnancy following treatment for breast cancer, in a cohort of women from Victoria, Australia, with a first diagnosis of invasive breast cancer | Wrong outcomes |
| Wang 2021 | Maternal and Fetal Outcomes of Acute Leukemia in Pregnancy: A Retrospective Study of 52 Patients | Wrong outcomes |
| Terzi 2010 | Surgical treatment of a gastric cancer in a pregnant woman without performing abortion | Wrong outcomes |
| Sonego Gomes 2022 | Living with pregnancy-associated cancer: grounded theory based on family experiences | Wrong patient population |
| Castillo-Luna 2017 | Ovarian cancer in the pregnancy: An institutional experience and literature review | Wrong patient population |
| Jeppesen 2011 | Successful Twin Pregnancy Outcome After In Utero Exposure to FOLFOX for Metastatic Colon Cancer: A Case Report and Review of the Literature | Wrong outcomes |

**Supplementary Table 4: Synthesised Findings Table**

| **Findings** | **Categories** | **Synthesised findings** |
| --- | --- | --- |
| F1, F2, F3, F6, F8, F9, F10, F11, F16, F17, F18, F19, F20, F26, F28, F29, F30, F33, F38, F43, F46, F49, F50, F51 | Distress, overwhelm and fear  Experience of cancer and pregnancy  Concerns for impact on baby, children and family  Existential distress  Advocacy and strength | Psychological impact  Women’s hopes and fears, distress, dyadic health and wellbeing, overwhelming emotions, reflections on spirituality. |
| F4, F5, F13, F14, F24, F31, F34, F36, F37, F44, F45, F48 | Feminine identity  Reshaping of Motherhood  Breastfeeding  Future fertility | Woman’s identity  Women’s identity, ‘sense of self’, expectations, breastfeeding and future fertility . |
| F7, F12, F15, F21, F22, F23, F25, F27, F32, F35, F39, F40, F41, F42, F43, F47 | Specialist care – coordination, integrating, conflicts, and planning  Decision making and information sharing  Supports – partners and family; support groups  Neonatal care – including terminations and concerns for baby | Complex care  Specialist care, multidisciplinary coordination and supports. |
